# Double Feature: Carcinoma and Sarcoma Present in a Single Breast Tumor

**DOI:** 10.1155/2012/232851

**Published:** 2012-11-05

**Authors:** Catherine M. Stefaniuk, Timothy Jones

**Affiliations:** Department of General Surgery and Department of Family Medicine, West Virginia School of Osteopathic Medicine, 400 North Lee Street, Lewisburg, WV 24901, USA

## Abstract

*Introduction*. Primary breast sarcomas (PBSs) are rare nonepithelial breast tumors compromised of mesenchymal mammary tissue. Although its rare nature has made the best mode of PBS treatment difficult to determine, it seems better to treat it more like a sarcoma creating clear negative margins verses breast carcinoma utilizing lumpectomy, partial mastectomy, and total mastectomy. *Case*. A 47-year-old obese Caucasian postmenopausal female G2P2 presents with a breast lump demonstrating a histological sample with a biphasic pattern consistent with both ductal carcinoma containing typical malignant epithelial cells and sarcomatous differentiation of carcinosarcoma. *Conclusion*. Carcinosarcoma is a rare breast malignancy. Sarcomas of the breast tend to be negative for estrogen receptor and lack known risk factors. Current recommended treatment is to treat breast sarcomas like other soft tissue sarcomas by performing wide local excision instead of partial mastectomy. Antiestrogens and other chemotherapeutic agents typically used in breast epithelial malignancies are not recommended since these sarcomas tend to be negative with these receptors.

## 1. Introduction

Primary breast sarcomas (PBSs) are rare nonepithelial breast tumors compromised of mesenchymal mammary tissue [1]. PBS was originally described in 1887 with <1,000 cases referenced in the medical literature since the 19th century [1]. Currently, it is still undetermined what is the best treatment modality for patients presenting with breast sarcoma. There are seven different groupings of PBS which include leiomyosarcoma, carcinosarcoma, angiosarcoma, epithelioid cell sarcoma, and rhabdomyosarcoma [1]. In one retrospective study of PBS from 1986 to 2006 [1], 13 patients were studied. Patients underwent either partial mastectomy or total mastectomy with patients that had axillary lymph node removal showing negative pathological results. It appears that metastatic transit by axillary lymph nodes does not occur though it is debatable whether or not to do sentinel lymph node sampling [1–4]. Tumor size has been shown to be a key variable that is significantly associated with 5-year survival rates and risk of local recurrence; tumors > 5 cm have a poor prognosis while <5 cm have better outcomes [1–3]. Negative surgical margins have been shown to be key for local recurrence and overall survival [2–4]. Although its rare nature has made the best mode of PBS treatment difficult to determine, it is better to treat it more like a sarcoma that may be found elsewhere (clear negative margins) verses breast carcinoma (lumpectomy, partial mastectomy, and total mastectomy) [1, 3, 4]. Neoadjuvant chemotherapy and radiotherapy are recommended to treat micrometastatic disease especially if tumor size is larger than 5 cm^2^. PBS tends to be negative for estrogen receptor, progesterone receptor, and Her2 mutations therefore making hormone therapy ineffective as an adjuvant [3]. In this paper, we discuss our findings and surgical procedure to provide more cases that will aid in determining an outline for how to treat patients with PBS in the future.

## 2. Case

A 47-year-old obese caucasian postmenopausal female G2P2 with a history of diabetes, hyperlipidemia, anemia, arthritis, tobacco abuse, and asthma presented with a left upper inner-quadrant breast lump. She denied any first degree family medical history of cancer of any type. Family history was positive for diabetes, asthma, heart disease, chemical dependency, hypertension, and kidney disease. Patient had an abnormal mammogram which illustrated a mass in the left breast at the 11 o'clock position in the upper inner quadrant that has an abnormal asymmetric density rounded with microlobulated margin. Her previous mammogram was 5 years prior.

She was referred to general surgery for evaluation. Upon breast exam, it was noted that the left breast was larger than the right with patient supine. Right breast was slightly more nodular than left without any dominant mass or lesion appearing normodense for her age. Her left breast revealed an increased dense area that was about 2 cm × 2 cm in upper inner quadrant approximately 10 cm from the areola in the 11 o'clock position. Margins were vaguely palpable. Mobile nonenlarged lymph node was noted in the left axilla. All other findings on physical examination were noncontributory. We pursued her condition by performing an ultrasound-guided biopsy of the irregular-shaped mass located in the 11 o'clock position. Several specimens were taken using SenoRx EnCor system, and specimens were sent to pathology. Pathology results indicated malignant carcinosarcoma and invasive ductal carcinoma.

## 3. Cytologic Microscopic Description

Specimen preparation included 2 Pap stains and 2 Wright-Giemsa stains. On analysis, individual malignant cells were visualized with significant nuclear pleomorphism enlarged irregular granular nuclei, and minimal cytoplasm with occasional mitotic figures. Sample demonstrated a biphasic pattern consistent with both ductal carcinoma with more typical malignant epithelial cells and sarcomatous differentiation consistent with carcinosarcoma. There were portions of the tumor showing significant pleomorphic nuclei with numerous multinucleated cells with prominent eosinophilic nucleoi with fluffy and spindle-shaped nuclei and atypical mitoses. 

Immunoperoxidase stains were performed producing positive results for actin consistent with sarcomatous component and negative for both estrogen and progesterone receptor. On biopsy, no definitive evidence of lymphovascular invasion, carcinoma *in situ*, or microcalcifications was identified. HER2 : chromosome 17 (D17Z1) ratio analysis was then performed which showed negative HER2 gene amplification with an HER2 : D17Z1 equal to 1.17. 

## 4. Surgical Procedure

In accordance with current evidence based guidelines [4–6], we performed a partial mastectomy/quadrantectomy. Prior to surgery, patient was prepped by radiology for a sentinel lymph node biopsy with frozen section analysis approximately two hours before her operation with radioactive tracer. Isobars were marked on the axillary area and nuclear medicine mapping was used to locate the sentinel node. The sentinel node was identified in the midaxillary line, elevated, and removed. Nuclear scanning was retested over the area and shown to be negative. Pathologist confirmed lymphocytes in the node and did not note metastasis. If the sentinel node was located substernally, it would have been left in place and targeted for radiation therapy. If sentinel node along midaxillary line was positive, a partial mastectomy of the whole breast with axillary lymph nodes would have been removed. Since the sentinel node was negative, a partial mastectomy/quadrantectomy was performed without removal of any further lymph nodes.

Preceding surgery, the palpable mass was marked in the upper medial quadrant of the left breast via ultrasound with generous margins drawn with pen. The mass was removed using a paddle of ellipsed skin at approximately 7 cm × 4 cm widening down to the pectoralis fascia with subtraction of fascia as well. A bovie was used set at 20 degrees to remove the breast mass and cauterize perforating vessels. Clips were placed along the area where the removed fascia was to tag previous location of the tumor. Incision was closed using vicryl for deep dermis and monocryl for skin. Dermabond was spread along the closure site. Patient has well tolerated the procedure, and positive cosmetic results were noted. No drain was inserted, and the patient had one post-op day in the hospital. 

Final pathology of the left axillary sentinel lymph node showed sinus histiocytosis with lymphoid hyperplasia without histologic evidence of malignancy. Anterior breast tissue showed adipose tissue with focal septal fibrosis lacking histologic malignancy. The lumpectomy illustrated poorly differentiated invasive ductal carcinoma with sarcomatous features. The Nottingham histologic grade: nuclear pleomorphism of 3, tubule formation of 3, and mitotic count of 3 (22/10HPF) made the combined Nottingham score 9. The breast lumpectomy size was 10.1 × 6.0 × 7.2 cm with irregular tumor mass measuring 2.3 × 2.0 × 1.8 cm at its greatest dimension. Tumor had no identified microcalcifications, lymphatic/vascular invasion, nor skin involvement. Venous vascular invasion was suspicious in one section on analysis. Surgical resection margins were free of tumor; tumor was approximately 1.5 cm of inferior resection margin, 3.0 cm on superior resection margin, 3.4 cm from peripheral resection margin, and 4.2 cm from medial resection margin. Pathologic state is IIA and TNM grading of pT2pN0pMX. Immunoperoxidase stains were performed and revealed a population of sarcomatous appearing cells with the tumor to be positive for actin; stains for estrogen and progesterone receptors were uniformly negative. Overlying skin and dermis was free of malignancy.

After surgery patient was referred to oncologist for followup. One year out from surgery patient is well with no signs of recurrence of sarcoma of the breast.

## 5. Commentary

Carcinosarcoma is a rare breast malignancy. Sarcomas of the breast tend to be negative for estrogen receptor and lack known risk factors [3]. Current recommended treatment is to treat breast sarcomas as other soft tissue sarcomas by performing wide local excision instead of partial mastectomy [3–5]. Anti-estrogens and other chemotherapeutic agents typically used in breast epithelial malignancies are not recommended due to the lack of efficacy since these sarcomas tend to be negative with these receptors [3]. In our patient, we performed a sentinel lymph node dissection to ensure that no metastasis to the draining lymph nodes had occurred due to the carcinomatous features of the breast. Since carcinosarcomas of the breast tend to follow a sarcomatous path [4]; we proceed with tumor removal with sizeable resection of the tumor margins. 

In addition to wide excision, it is also recommended to provide adjuvant radiotherapy to improve overall local control [4]. Adjuvant chemotherapy has been used with some success, but since response rates are limited, it is hard to recommend [4]. Current chemotherapeutics that have been used/recommended are doxorubicin alone or in combination with ifosfamide due to their use in other soft tissue sarcomas [3]. 

To ensure that no metastasis is present, a full metastatic work-up can be performed including but not limited to a chest X-ray and/or bone scan. 

We recommend to any surgeon presented with a similar patient that a thorough surgical plan is to be mapped out, and all options are to be considered. This way, both you and patient will be prepared for possibly different surgical procedures and know potential outcomes.
